# Predictive modeling for identification of older adults with high utilization of health and social services

**DOI:** 10.1080/02813432.2024.2372297

**Published:** 2024-07-03

**Authors:** Heba Sourkatti, Juha Pajula, Teemu Keski-Kuha, Juha Koivisto, Mika Hilvo, Jaakko Lähteenmäki

**Affiliations:** a VTT Technical Research Centre of Finland Ltd, Espoo, Finland; b Finnish Institute of Health and Welfare (THL), Helsinki, Finland

**Keywords:** Machine learning, predictive modeling, secondary use of data, health and social services usage, older adults

## Abstract

**Aim:**

Machine learning techniques have demonstrated success in predictive modeling across various clinical cases. However, few studies have considered predicting the use of multisectoral health and social services among older adults. This research aims to utilize machine learning models to detect high-risk groups of excessive health and social services utilization at early stage, facilitating the implementation of preventive interventions.

**Methods:**

We used pseudonymized data covering a four-year period and including information on a total of 33,374 senior citizens from Southern Finland. The endpoint was defined based on the occurrence of unplanned healthcare visits and the total number of different services used. Input features included individual’s basic demographics, health status and past usage of healthcare resources. Logistic regression and eXtreme Gradient Boosting (XGBoost) methods were used for binary classification, with the dataset split into 70% training and 30% testing sets.

**Results:**

Subgroup-based results mirrored trends observed in the full cohort, with age and certain health issues, e.g. mental health, emerging as positive predictors for high service utilization. Conversely, hospital stay and urban residence were associated with decreased risk. The models achieved a classification performance (AUC) of 0.61 for the full cohort and varying in the range of 0.55–0.62 for the subgroups.

**Conclusions:**

Predictive models offer potential for predicting future high service utilization in the older adult population. Achieving high classification performance remains challenging due to diverse contributing factors. We anticipate that classification performance could be increased by including features based on additional data categories such as socio-economic data.

## Introduction

The aging population causes a growing need for health and social services and the related costs are increasing rapidly. At the same time, there is an increasing shortage of professional workforce. Based on earlier research, a small amount of healthcare customers consumes the majority of healthcare services [1]. In Finland, 10% of population consumes 80% of all costs incurred in health and social services [2]. Furthermore, a specific subgroup of these ‘heavy users’ are users of multi-sectoral services consuming a large number of different health and social services [2]. These individuals with increased need for multi-sectoral services are not always recognized properly in health and social services settings although they would benefit from integrated care and coordinated preventive interventions. Early identification of individuals with increased need for multi-sectoral services would enable preventive actions having a positive impact on quality of life and costs of health and social services.

Data-driven prediction of health and social services usage has been proposed in several earlier studies. Typically, the underlying goal has been to provide information needed to improve efficiency and quality of healthcare processes. Most cases involve prediction of hospital readmissions and emergency department visits (e.g. [3–8]). Examples of other addressed areas include exacerbation of chronic disease patients (e.g. [9,10]), future diagnoses (e.g. [11]), hospital no-show appointments (e.g. [12]) and unmet need for social care [13]. Several approaches are focused on the prediction of healthcare costs [14].

While most of the existing studies focus on predicting health risks of patients, a minority of earlier studies have addressed the overall use of health and social services among older adults. Bardsley et al. [15] and Nakubulwa et al. [16] present approaches for predicting, which individuals are expected to begin receiving intensive social care during the next 12 months. Bardsley et al. [15] define intensive social care by the occurrence of one of three events: admission to care home, start of intensive home care or social care costs exceeding a predefined threshold [15]. Correspondingly, Nakubulwa et al. define the predicted endpoint as the start of home care visits or admission to care home [16]. Stafford et al. analyze the effect of household co-residents on the use of health and social services by older adults with multiple long-term conditions [17].

Complementing the earlier approaches, the objective of the MAITE study (‘Data-driven identification of elderly individuals with future need for multi-sectoral services’) was to predict the risk of high health and social services utilization at early stage – well before the start of intensive home care or admission to elderly home. In particular, the goal was to develop explainable machine learning models to identify older adults at risk of excessive service use. Such models would enable individuals at risk to be invited for preventive light-weight interventions designed to avoid accumulation of social and health related problems. For model development, we used retrospective patient and customer data (real-world data) recorded in a public regional health and social services setting in Finland.

## Methods

### Data

We carried out a retrospective study using data from the health and social data registers of the wellbeing services county of Päijät-Häme, which is a public provider of social services, primary and secondary healthcare services for a catchment area of 206,000 inhabitants in southern Finland. The study participants represented nine municipalities of the region: Lahti, Hollola, Orimattila, Asikkala, Iitti, Kärkölä, Padasjoki, Hartola and Myrskylä. Two of the Päijät-Häme municipalities (Heinola and Sysmä) were excluded, since full register data from them were not available in the Päijät-Häme data lake.

We used individual-level data from customer and patient registers of Päijät-Häme under the national legislation on secondary use of health and social data. The data cover years 2018–2021 documenting demographics, vital status, healthcare and social services visits, social service benefits decisions and physical function assessments. We obtained the data permit from the wellbeing services county of Päijät-Häme in March 2022. The data were made available for researchers in pseudonymized form in the secure processing environment of Findata (Finnish Data Permit authority) in July 2022.

### Modeling approach

**Figure 1.**
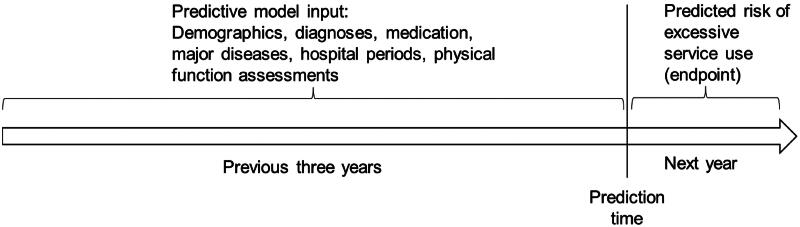
Study setting.

The basis of the prediction model was established in joint workshops where Päijät-Häme elderly services personnel contributed to the endpoint and feature selection. Additionally, Päijät-Häme data analysts and IT personnel contributed by knowledge on the data resources available for the model development. The inclusion criteria were defined as: (1) permanent residence in the Päijät-Häme region during years 2018–2021, (2) age 70–90 years (on 1 January 2022), (3) usage of the health and social services of Päijät-Häme at least once in year 2018. Participants, who died during the years 2018–2021 were excluded. Our modeling objective was to use the data from three consecutive years for predicting the risk of high utilization of health and social services in the fourth year (Figure 1).

We defined the study endpoint (high utilization of health and social services) by the following coexistent conditions:

- Occurrence of one or more unplanned healthcare service visits in year 2021.
- Number of unplanned visits was higher in year 2021 than the average of years 2018–2020.
- Usage of two or more different health and social services in year 2021.
- Number of used services of different types was higher in year 2021 than the average of years 2018–2020.

The categorization of health and social services was based on the service classification published in the national code server [18]. They include primary and specialized outpatient clinic, hospital visits, mental health services, substance abuse services and home care services. Unplanned healthcare visits are visits to a hospital or health center that are not based on a scheduled appointment.

### Variables

We first constructed a set of 28 features expected to be important based on earlier research [6,15,16] as well as the information received from the Päijät-Häme elderly services personnel. All features were based on data from years 2018–2020. The number of features was reduced to 18 after removing 10 overlapping features to ensure that maximum correlation coefficient between any pair of features in the training set was smaller than 0.5 among all participants. The selected features are listed in Table 1. They include basic demographics (sex, age and living area), existing diseases (mental health condition, diabetes, musculoskeletal condition, hypertension and injury) and the total number of major diseases. The included major disease groups were selected based on the morbidity index of the Finnish Institute of Health and Welfare (THL) [19]. We also selected features related to various types of healthcare resources usage (unplanned visits, hospital periods and visits for health examinations and advisory services) and usage of specific medication groups: anti-inflammatory or antirheumatic medication and psycholeptics or psychoanaleptics. These medication groups were selected because they are largely used among the older adults and their use involves a high risk for adverse drug reactions [20–22]. The Resident Assessment Instrument (RAI) [7] results were included only for the home care group, for which the assessments have been carried out consistently. Most of the features are binary. Age, number of major diseases, number of hospital visits and ADLH-6/CPS-6 (Activities of Daily Living Hierarchy/Cognitive Performance Scale) RAI indicators are numeric. Numeric variables were normalized by dividing their deviation from mean value by the standard deviation. More information about the used RAI indicators can be found in Supplementary Table S1.

**Table 1.** Model features.

	Variable type	ICD-10 code/description
Sex	Binary	
Age	Numeric	Age in years on 1 January 2022
Living in city	Binary	Living address in the city of Lahti during 2018–2020
Diabetes	Binary	[E10–E14, G59.0, G63.2, H28, H36.0, I79.2, M14.2, M14.6, N08.3]^a^
Hba1c out of range	Binary	Hba1c laboratory result out of recommended range 20–42 mmol/mol
Glucose out of range	Binary	Glucose laboratory result out of recommended range 4–6 mmol/l
HbA1c not tested	Binary	HbA1c laboratory test has not been made
Glucose not tested	Binary	Glucose laboratory test has not been made
Dementia	Binary	[F00–F03, G30, F05.1, F10.73, F11.73, F14.73, F16.73, F18.73, F19.73]^a^
Mental health	Binary	F00–F99^a^
Musculoskeletal condition	Binary	M00–M99^a^
Injury	Binary	S00–T78^a^
Hypertension	Binary	I10^a^
Advisory services used	Binary	Z70–Z76^a^
Infection risk identified	Binary	Z20–Z29^a^
Health examination	Binary	Z00–Z13^a^
Medication (group M01)	Binary	[Anti-inflammatory or antirheumatic medication (ATC M01)]^b^
Medication (groups N05/N06)	Binary	[Psycholeptics (ATC N05) or psychoanaleptics (ATC N06)]^b^
Number of major diseases	Integer	Number of major diseases in 2018. Major diseases: cardiovascular disease, cancer, musculoskeletal condition, mental health condition, chronic respiratory disease and diabetes.
Unplanned visits	Binary	One or more unplanned visits in 2020 AND number of unplanned visits in 2020 higher than the average of years 2018–2019.
No dental visits	Binary	No visits to public dental health services in years 2018–2020.
Hospital visits	Numeric	Number of hospital periods in 2018–2020
Daily living performance	Numeric	[RAI assessment^c^: ADLH-6 (Activities of Daily Living Hierarchy). Deviation from mean score.
Cognitive performance	Numeric	RAI assessment^c^: CPS-6 (Cognitive Performance Scale). Deviation from mean score.
Fallrisk	Binary	RAI assessment^c^: MAPLe (method for assigning priority levels), fallrisk
Nutrition problem	Binary	RAI assessment^c^: MAPLe (method for assigning priority levels), nutrition problem

RAI: resident assessment instrument; ATC: anatomical therapeutic chemical; ICD: International Classification of Diseases.

^a^ One of the ICD-10 codes recorded in 2018–2020.

^b^ Medication prescribed at least once in 2018–2020.

^c^ Physical function assessment performed in 2018–2020 (only for home care customers).

### Analyses

We first processed the raw input data files to compute the features and the endpoint status for each participant. We used two methods for binary classification: logistic regression and eXtreme Gradient Boosting (XGBoost). XGBoost is widely recognized for its performance in dealing with complex datasets and its capacity to capture non-linear relationships between features. Besides XGBoost, we also used logistic regression which is a linear model and could capture linear relationships. This approach enabled us to compare models across two different types of variable relationships. For both methods, we divided the data set into two parts: 70% for model training and 30% for model testing. We stratified the training and testing sets based on endpoint occurrence and the following features: dementia, diabetes, mental health condition, number of major diseases, sex and age. The objective of the stratification was to ensure similar features distributions in both training and test sets, and hence reduce bias.

We optimized the XGBoost model by tuning the hyperparameters such as tree depth, learning rate, ensemble size, split loss (gamma), scale positive weights, features and training samples subsampling using stratified fivefold cross-validation on the training data [23,24]. Compared to logistic regression XGBoost is inherently less interpretable due to its ensemble nature and complexity. In order to interpret its non-linear results, we used SHapley Additive exPlanations (SHAP) on the training data to reveal the effect of individual features on the model’s prediction [25]. SHAP values provide insights to the relative importance of each feature by assigning a numerical value to its impact on the predicted outcome. We report relative importance of each feature by listing its SHAP ranking.

For assessing the performance of our binary classifiers, we used the area under the curve (AUC), sensitivity and positive predictive value (PPV). We reported the sensitivity and PPV corresponding to the false positive rate of 25%, which we estimated to be a tolerable level in operational use of the model.

### Subgroups

We trained and tested the model with the full cohort and with six subgroups of interest: dementia, diabetes, mental health, no major disease, elderly services customer and home care customer (Supplementary Table S2). The subgroups are partly overlapping: in particular, all participants with dementia belong also to the mental health subgroup. Analysis by subgroups was considered appropriate, since the actual need for services considerably varies between the groups. The no major disease group included individuals who did not have any of the six major diseases listed in Table 1. We considered an individual to be an elderly services customer if the individual had a decision on elderly services benefit or if the individual had used home care services during the period 2018–2020. We formed the target groups based on the inputs provided by the Päijät-Häme personnel.

## Results

Characteristics of the study participants per target group are shown in Table 2. Altogether 33,374 participants fulfilled the inclusion criteria, which allowed a training set of 23,374 and test set of 10,000 participants for the full cohort. Combining training and test sets, 12.9% of participants achieved the endpoint. The endpoint occurrence was lowest in the group with no major diseases (7.9%) and highest in the home care customer group (16.7%).

**Table 2.** Characteristics of study participants.

	All participants	Dementia	Diabetes	Mental health	No major disease	Elderly services customer	Home care customer
Number of participants	33,374	2169	6358	4676	5793	4181	2320
Training set size	23,374	1516	4445	3265	4106	2914	1623
Test set size	10,000	653	1913	1411	1687	1267	697
Sex, female, *n* (%)	19,089 (57.2%)	1354 (62.4%)	3089 (48.6%)	2946 (63.0%)	3348 (57.8%)	2690 (64.3%)	1549 (66.8%)
Age, years, mean (SD)	77.4 (5.4)	82.1 (5.2)	77.6 (5.3)	78.8 (5.8)	76.0 (5.0)	81.1 (5.8)	81.9 (5.7)
Endpoint (full cohort), *n* (%)	4308 (12.9%)	317 (14.6%)	929 (14.6%)	758 (16.2%)	458 (7.9%)	681 (16.3%)	388 (16.7%)
Endpoint (training set), *n* (%)	3018 (12.9%)	222 (14.6%)	668 (15.0%)	526 (16.1%)	308 (7.5%)	482 (16.5%)	280 (17.3%)
Endpoint (test set), *n* (%)	1290 (12.9%)	95 (14.5%)	261 (13.6%)	232 (16.4%)	150 (8.9%)	199 (15.7%)	108 (15.5%)
Dementia, *n* (%)	2169 (6.5%)	2169 (100%)	494 (7.8%)	1554 (33.2%)	0 (0%)	1289 (30.8%)	954 (41.1%)
Diabetes, *n* (%)	6358 (19.1%)	494 (22.8%)	6358 (100%)	1087 (23.2%)	0 (0%)	1127 (27.0%)	684 (29.5%)
Mental health, *n* (%)	4676 (14.0%)	1554 (71.6%)	1087 (17.1%)	4676 (100%)	0 (0%)	1740 (41.6%)	1162 (50.1%)
Number of major diseases, mean (SD)	1.03 (0.85)	1.47 (0.93)	1.88 (0.84)	1.41 (0.92)	0.0 (0.0)	1.50 (0.94)	1.57 (0.96)

SD: standard deviation.

### Factors affecting risk for high service utilization

The logistic regression coefficients and XGBoost SHAP rankings are listed in Table 3 for the full cohort and in Supplementary Table S3 for all subgroups. SHAP summary plots are shown in Supplementary Figure S1(a–g). The list of the optimized hyperparameters for XGBoost is shown in Supplementary Table S4.

**Table 3.** Logistic regression coefficients (related *p* values) and XGBoost SHAP ranking.

Feature	Logistic regression (*p* value)	XGBoost SHAP ranking (direction)
Sex	−0.031 (0.453)	16 (–)
Age	0.241 (<0.001)	1 (+)
Living in city	−0.289 (<0.001)	4 (–)
Diabetes	0.024 (0.679)	14 (+)
Dementia	−0.243 (0.006)	18 (–)
Mental health	0.222 (<0.001)	10 (+)
Musculoskeletal condition	0.161 (0.001)	5 (+)
Injury	0.122 (0.006)	11 (+)
Hypertension	0.008 (0.860)	17 (+)
Advisory services used	0.144 (0.008)	9 (+)
Infection risk identified	−0.017 (0.691)	13 (+)
Health examination	0.009 (0.834)	12 (+)
Medication (group M01)	0.065 (0.147)	8 (+)
Medication (groups N05/N06)	0.169 (<0.001)	7 (+)
Number of major diseases	0.138 (<0.001)	2 (+)
Unplanned visits	0.303 (<0.001)	3 (+)
No dental visits	0.052 (0.266)	15 (+)
Hospital visits	−0.212 (<0.001)	6 (–)

SHAP: SHapley Additive exPlanations.

SHAP ranking: smaller ranking indicates higher importance of a feature. Sign refers to the direction of the effect.

According to the analysis of all participants together, age is a positive predictor for high service utilization as expected. Some health problems (mental health except dementia patients, musculo-skeletal conditions and injuries) are also positive predictors for the endpoint. Other positive predictors are unplanned visits, number of major diseases and usage of psycholeptics or psychoanaleptics. Number of hospital periods and living in the urban location show up as a factor decreasing the risk for high service utilization. Also, living in city area predicts a decreased risk for high utilization of services.

The subgroup-based results indicate similar trends with the full cohort, although statistical significance is not always achieved due to a smaller sample size. In the group with no major disease, the use of anti-inflammatory or antirheumatic medication predicts high service utilization. In the home care group, the RAI assessments (ADLH-6 and CPS-6) are negative predictors of high service use.

### Classification performance

Classification performance results for logistic regression and XGBoost methods are shown in Table 4. For the full cohort, the AUC was 0.61 for both methods. While there are some differences between subgroups, a similar level of performance is achieved with both methods. The AUC values are slightly higher for the full cohort and the group with no major disease when compared to the disease-specific groups.

**Table 4.** Prediction model performance.

	All	Dementia	Diabetes	Mental health	No major disease	Elderly services customer	Home care customer
Logistic regression							
AUC	0.61	0.57	0.57	0.59	0.61	0.58	0.62
Sensitivity, 25% FPR	0.39	0.33	0.36	0.38	0.36	0.34	0.39
PPV, 25% FPR	0.19	0.18	0.18	0.23	0.12	0.20	0.22
XGBoost							
AUC	0.61	0.61	0.55	0.57	0.62	0.57	0.58
Sensitivity, 25% FPR	0.39	0.32	0.30	0.32	0.38	0.32	0.31
PPV, 25% FPR	0.19	0.18	0.16	0.20	0.12	0.19	0.19

AUC: area under the curve; FPR: false positive rate; PPV: positive predictive value.

## Discussion

We have presented an approach for identifying older adults in risk of excessive use of health and social services based on individual’s past usage of services and current health status.

Our prediction models achieved classification performance of 0.61 (AUC) in the full cohort while the results varied between 0.55 and 0.62 (AUC) in the subgroup analysis. Age, earlier unplanned visits, number of major diseases, mental health conditions (except dementia), musculoskeletal conditions, injuries and usage of psycholeptics or psychoanaleptics were most significant predictors for future excessive service usage. Hospital visits, dementia and living in the city were predictors for less service usage. In the home care, customer subgroup declined daily living and cognitive performance predicted less service use. In the subgroup with no major disease, the use of anti-inflammatory or antirheumatic medication predicts high service utilization.

In general, our results show similar trends as observed in other studies [15,16]. For example, the increasing effect of age on the need for services is evident based on our study and others. The finding that individuals living in the city had smaller risk to high service utilization may be related to the higher income level. The average income level in the regional center (Lahti) is about 9% higher than in the surrounding eight municipalities [26]. This suggests similar association between deprivation and need for social services as observed in earlier studies [16].

The classification accuracy of our prediction model is relatively low reflecting the fact that a high variety of contributing factors affect future service usage. Similar performance resulted from earlier research targeted to predict risk of unplanned hospital admission of home care clients based on RAI data [7]. Better level of classification performance (AUC >0.7) was reported by Nakubulwa et al. [16] in predicting the citizen’s need for adult social care.

Socioeconomic factors and emergency admissions have earlier been found to be associated with social services use [16]. Such data were not available in our case, which may partly explain the difference in classification performance. Additional reason for the performance difference may be that in the earlier studies of Bardsley et al. and Nakubulwa et al. the end point was more clearly defined based on the start of using homecare or elderly housing services [15,16]. In our case, the endpoint was more broadly based on large and increasing services usage, aiming to capture individuals with social and health problems at earlier stage.

In our approach, one challenge is to define the end point in a way that captures only unnecessary and avoidable use of services. Number of health and social services visits alone is not a feasible endpoint, as for example, the management of chronic diseases often involves frequent control visits, which are necessary and aligned with the recommended treatment practice. To overcome this challenge, we defined the endpoint based on unplanned healthcare visits instead of all visits and we also required an increasing trend to take place in service usage. Thus, individuals with high and justified need for services were not considered to be individuals at risk if the usage of services was planned or not increasing. We also carried out the analysis per subgroup, which enabled us to predict individual’s risk in relation to a group with roughly similar background and need for services.

Our classification approach began with a baseline logistic regression model and progressed to the implementation of the more robust XGBoost method in pursuit of enhanced performance [27]. Despite the improvements achieved through optimizing the XGBoost model, the results remain comparable to those obtained by the logistic regression model. This parallel performance suggests the possibility of lacking informative features, potentially limiting the full utilization of the XGBoost model’s capabilities. The results suggest a tendency towards linearity in the association between the features and the endpoint making the utilization of a more complex model like XGBoost unnecessary.

We included diabetes as a potential factor affecting the risk of high service utilization. In contrary to the finding by Nakubulwa et al. [16], we could not find such association. Furthermore, within the diabetes group out-of-range or missing laboratory tests of glycated hemoglobin (HbA1c) and glucose were not predictors of high service utilization.

RAI assessment results for daily living and cognitive performance showed that low scores (good performance) were associated with higher risk for high service utilization. Thus, individuals already consuming the homecare services may increase service usage only if they are in relatively good physical and mental condition. Similar effect is seen when observing individuals with dementia and those with high amount of hospital periods: they seem to be in less risk of high service utilization. This observation reflects our original objective of identifying the individuals at early stage. Applying the model to homecare customers or hospitalized individuals seems not to be feasible. From the prevention perspective, the group of individuals with no major disease seems to be highly relevant. The mean age in this subgroup was 76.0 years, while in the dementia and home care customer groups the mean age was 82.1 and 81.9 years, respectively. Identifying healthy older adults with risk of high service utilization in the future would enable preventive actions, such as inviting those individuals to health examinations, physical function tests and lifestyle interventions.

The limitations of our study are related to the availability of data in registers of the health and social care provider. In addition to socio-economic factors [28] and emergency admissions referred above, also data concerning no-show appointments [12] and household co-resident context [17] would have been potential predictors of the endpoint, but were not available. Our data were limited to a period of four years, allowing the features to be constructed based on three years while the data from the fourth year was used for the endpoint. In reality, social and health problems accumulate during longer periods of time [13] and it seems likely that a longer period would allow us to create more accurate models. Also, the number of participants was not large enough to enable a more fine-grained sub-group analysis. Finally, the available data set may have included individuals, who moved away from the Päijät-Häme region during the study, which may cause a small bias to the analysis. Based on Statistics Finland, about 0.5% of population in this age group moves out from the county yearly.

Even with the limitations, we expect that our predictive models could be beneficial for focusing preventive interventions to appropriate target groups. When applied in such context the limited classification performance of the models can be tolerated as prediction errors will not directly lead to health hazards. Before deployment in operational health and social services, improvement of the classification performance would be necessary. We anticipate that the classification performance of the model could be improved by using socio-economic factors such as income, wealth and education as predictors.

## Conclusions

We used real world health and social data for developing models for predicting future high service utilization among older adults. Such models are expected to be useful for identifying individuals, which could benefit from targeted preventive interventions. Our models are explainable and enable investigation of features affecting the risk for high service usage. The achieved classification performance of the models is of the order of 0.6 (AUC). We anticipate that the classification performance can be improved by using additional data categories such as socio-economic data.

## Supplementary Material

Supplemental Material
