# Supplementary material for: Predictive modeling for identification of older adults with high utilization of health and social services
Source: Scand J Prim Health Care. 2024 Jul 3;42(4):609–16. doi: 10.1080/02813432.2024.2372297 (PMC11552250; doi:10.1080/02813432.2024.2372297)
Supplement: Supplemental Material [file IPRI_A_2372297_SM3448.docx]

Supplementary Table S1. RAI indicators assessed for home care customers.

| Indicator | Scale | Comment |
| --- | --- | --- |
|  |  |  |
| Activities of Daily Living Hierarchy (ADLH-6) | 0 - 6 | Assesses mobility within the unit, dining, use of the restroom, and personal hygiene.  0: Independent, 1: Supervision, 2: Limited, 3: Extensive, 4: Maximal, 5: Dependent, 6: Total dependent. |
| Cognitive Performance Scale (CPS-6) | 0 - 6 | Assesses short-term memory, comprehension, decision-making ability, level of consciousness, and ability to eat independently.  0: Intact, 1: Borderline Intact, 2: Mild Impairment, 3: Moderate Impairment, 4: Moderately Severe Impairment, 5: Severe Impairment, 6: Very Severe Impairment. |
| Method for Assigning Priority Levels (MAPLe), Fallrisk | yes/no | Question on fallrisk in the service need assessment based on the MAPLe method. |
| Method for Assigning Priority Levels (MAPLe), Nutrition problem | yes/no | Question on nutrition deficiency in the service need assessment based on the MAPLe method. |
|  |  |  |

**Abbreviations**: ADLH, Activities of Daily Living Hierarchy; CPS, Cognitive Performance Scale; MAPLe, Method for Assigning Priority Levels

Supplementary Table S2. Division of participants into subgroups

| Subgroup | Description |
| --- | --- |
| Dementia | At least one of the following ICD-10 codes recorded in years 2018-2020: F00-F03, G30, F05.1, F10.73, F11.73, F14.73, F16.73, F18.73, F19.73 |
| Diabetes | At least one of the following ICD-10 codes recorded in years 2018-2020: E10–E14, G59.0, G63.2, H28, H36.0, I79.2, M14.2, M14.6, N08.3 |
| Mental health | At least one of the following ICD-10 codes recorded in years 2018-2020: F00-F99 |
| No major disease | All participants , which did not have any of the following diseases (none of the corresponding ICD-10 codes recorded in 2018-2020): cardiovascular disease (I00-I99), cancer (C00-96, D06, D09.0-1, D30, D32-33, D41-43, D45-D47, D76, N87.2), respiratory disease (J23-J99), musculoskeletal disease (M00–M99), mental health condition (F00-F99), dementia (F00-F03, G30, F05.1, F10.73, F11.73, F14.73, F16.73, F18.73, F19.73) or diabetes (E10–E14, G59.0, G63.2, H28, H36.0, I79.2, M14.2, M14.6, N08.3) |
| Elderly services customer | All participants which had at least one documented home care visit or a positive elderly service benefit decision in 2018-2020 |
| Home care customer | All participants which had at least one documented home care visit in 2018-2020 |

**Abbreviations:** ICD, International Classification of Diseases

Supplementary Table S3. Logistic regression coefficients (related p-values) and XGBoost SHAP ranking for all participants and six subgroups. SHAP ranking: smaller ranking indicates higher importance of a feature. Sign refers to the direction of the effect.

|  | All participants | | Dementia | | Diabetes | | Mental health or substance abuse | | No major disease | | Elderly services customer | | Home care customer | |
| --- | --- | --- | --- | --- | --- | --- | --- | --- | --- | --- | --- | --- | --- | --- |
|  | LR ꟗ (p-value) | XGBoost SHAP ranking (direction) | LR ꟗ (p-value) | XGBoost SHAP ranking (direction) | LR ꟗ (p-value) | XGBoost SHAP ranking (direction) | LR ꟗ (p-value) | XGBoost SHAP ranking (direction) | LR ꟗ (p-value) | XGBoost SHAP ranking (direction) | LR ꟗ (p-value) | XGBoost SHAP ranking (direction) | LR ꟗ (p-value) | XGBoost SHAP ranking (direction) |
| Sex | -0.031 (0.453) | 16(-) | -0.189 (0.219) | 10(-) | -0.044 (0.613) | 14(+) | -0.147 (0.148) | 7(-) | -0.275 (0.025) | 6(-) | -0.078 (0.474) | 8(-) | -0.190 (0.195) | 19(-) |
| Age | 0.241 (<0.001) | 1(+) | 0.207 (0.009) | 3(+) | 0.206 (<0.001) | 2(+) | 0.219 (<0.001) | 3(+) | 0.284 (<0.001) | 1(+) | 0.135 (0.013) | 3(+) | 0.117 (0.110) | 2(+) |
| Living in city | -0.289 (<0.001) | 4(-) | -0.249 (0.134) | 4(-) | -0.498 (<0.001) | 1(-) | -0.332 (0.003) | 5(-) | -0.071 (0.598) | 11(-) | -0.188 (0.098) | 7(-) | 0.006 (0.971) | 16(-) |
| Diabetes | 0.024 (0.679) | 14(+) | 0.086 (0.666) | 9(+) | - | - | 0.120 (0.364) | 11(+) | - | - | 0.074 (0.570) | 12(+) | 0.042 (0.802) | 15(+) |
| Hba1c out of range | - |  | - |  | 0.092 (0.405) | 15(-) | - |  | - |  | - |  | - |  |
| Glucose out of range | - |  | - |  | 0.052 (0.672) | 9(+) | - |  | - |  | - |  | - |  |
| Hba1c not tested | - |  | - |  | 1.006 (0.430) |  | - |  | - |  | - |  | - |  |
| Glucose not tested | - |  | - |  | -0.149 (0.241) | 7(-) | - |  | - |  | - |  | - |  |
| Dementia | -0.243 (0.006) | 18(-) | - | - | -0.204 (0.260) | 16(+) | -0.298 (0.013) | 6(-) | - | - | -0.266 (0.049) | 16(-) | 0.043 (0.819) | 20(+) |
| Mental health | 0.222 (<0.001) | 10(+) | -0.023 (0.898) | 15(-) | 0.250 (0.054) | 8(+) | - | - | - | - | 0.078 (0.524) | 10(+) | 0.016 (0.921) | 11(+) |
| Musculoskeletal condition | 0.161 (0.001) | 5(+) | -0.064 (0.724) | 5(+) | 0.067 (0.528) | 12(+) | 0.077 (0.532) | 8(+) | - | - | -0.025 (0.845) | 15(-) | -0.134 (0.423) | 8(-) |
| Injury | 0.122 (0.006) | 11(+) | 0.165 (0.291) | 8(+) | 0.165 (0.291) | 9(+) | 0.100 (0.329) | 12(+) | -0.010 (0.921) | 8(+) | 0.052 (0.628) | 14(+) | 0.082 (0.564) | 14(+) |
| Hypertension | 0.008 (0.860) | 17(+) | 0.008 (0.860) | 14(-) | -0.041 (0.675) | 10(-) | -0.001 (0.993) | 14(+) | - | - | 0.085 (0.475) | 11(+) | 0.102 (0.524) | 18(+) |
| Advisory services used | 0.144 (0.008) | 9(+) | -0.094 (0.600) | 17(+) | 0.074 (0.482) | 11(+) | 0.150 (0.184) | 13(+) | 0.819 (<0.001) | 2(+) | 0.046 (0.690) | 17(+) | 0.044 (0.770) | 17(+) |
| Infection risk identified | -0.017 (0.691) | 13(+) | -0.069 (0.651) | 16(+) | 0.017 (0.854) | 13(+) | -0.042 (0.678) | 15(-) | -0.160 (0.186) | 9(-) | -0.010 (0.921) | 18(-) | -0.094 (0.495) | 22(-) |
| Health examination | 0.009 (0.834) | 12(+) | -0.178 (0.303) | 12(-) | 0.196 (0.025) | 6(+) | 0.042 (0.683) | 9(+) | 0.286 (0.065) | 4(+) | -0.227 (0.047) | 9(-) | -0.348 (0.028) | 9(-) |
| Medication (group M01) | 0.065 (0.147) | 8(+) | 0.034 (0.849) | 13(+) | -0.060 (0.545) | 17(-) | -0.128 (0.243) | 17(-) | 0.404 (0.014) | 5(+) | -0.264 (0.025) | 5(-) | -0.213 (0.180) | 13(-) |
| Medication (groups N05/N06) | 0.169 (<0.001) | 7(+) | -0.176 (0.460) | 6(-) | 0.172 (0.089) | 6(+) | -0.042 (0.744) | 16(-) | 0.558 (<0.001) | 3(+) | -0.117 (0.348) | 6(-) | -0.161 (0.386) | 5(-) |
| Number of major diseases | 0.138 (<0.001) | 2(+) | 0.169 (0.153) | 2(+) | 0.075 (0.218) | 3(+) | 0.044 (0.560) | 4(+) | - | - | 0.128 (0.106) | 4(+) | 0.138 (0.178) | 3(+) |
| Unplanned visits | 0.303 (<0.001) | 3(+) | 0.303 (<0.001) | 7(+) | 0.237 (0.010) | 4(+) | 0.450 (<0.001) | 2(+) | 0.228 (0.150) | 10(+) | 0.444 (<0.001) | 2(+) | 0.332 (0.020) | 6(+) |
| No dental visits | 0.052 (0.266) | 15(+) | 0.195 (0.285) | 11(+) | 0.074 (0.459) | 18(-) | 0.164 (0.160) | 10(+) | 0.012 (0.939) | 12(-) | 0.170 (0.178) | 13(+) | -0.143 (0.388) | 10(-) |
| Hospital visits | -0.212 (<0.001) | 6(-) | -0.456 (<0.001) | 1(-) | -0.251 (<0.001) | 5(-) | -0.360 (<0.001) | 1(-) | -0.004 (0.942) | 7(+) | -0.437 (<0.001) | 1(-) | -0.524 (<0.001) | 1(-) |
| Daily living performance | - |  | - |  | - |  | - |  | - |  | - |  | -0.200 (0.024) | 7(-) |
| Cognitive Performance | - |  | - |  | - |  | - |  | - |  | - |  | -0.225 (0.018) | 4(-) |
| Fall risk | - |  | - |  | - |  | - |  | - |  | - |  | 0.322 (0.252) | 21(+) |
| Nutrition problem | - |  | - |  | - |  | - |  | - |  | - |  | 0.357 (0.319) | 12(+) |

Supplementary Figure S1. SHAP summary plots

| 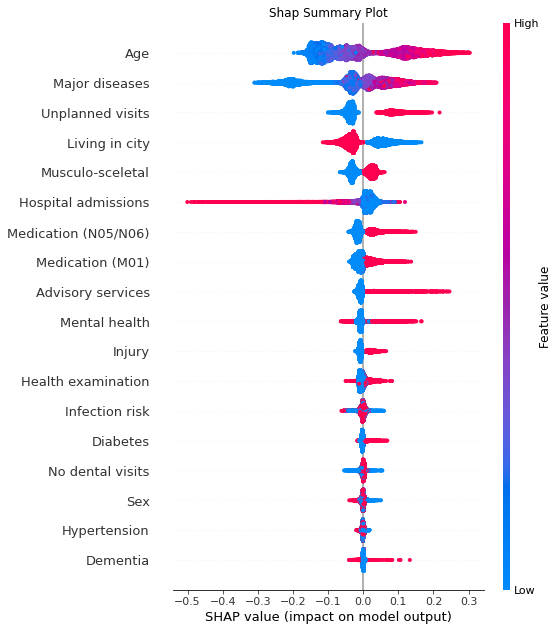  a: All participants. | 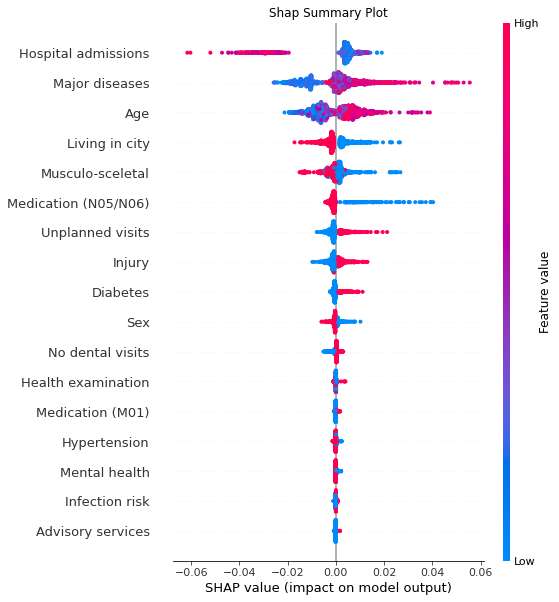  b: Dementia |
| --- | --- |
| 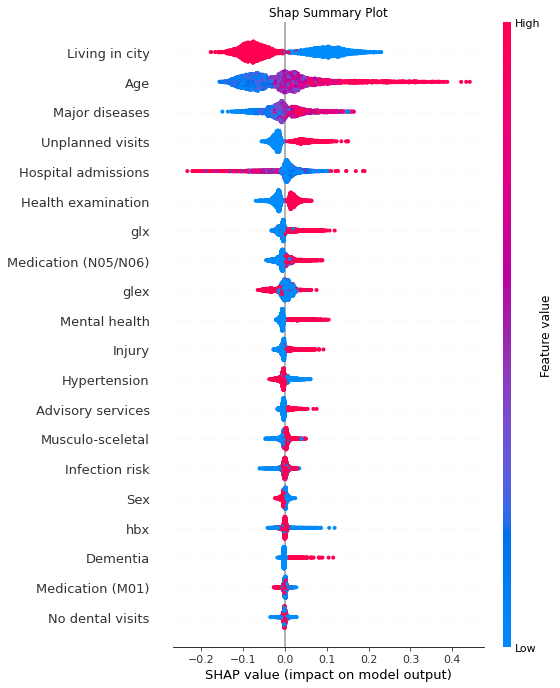  c: Diabetes | 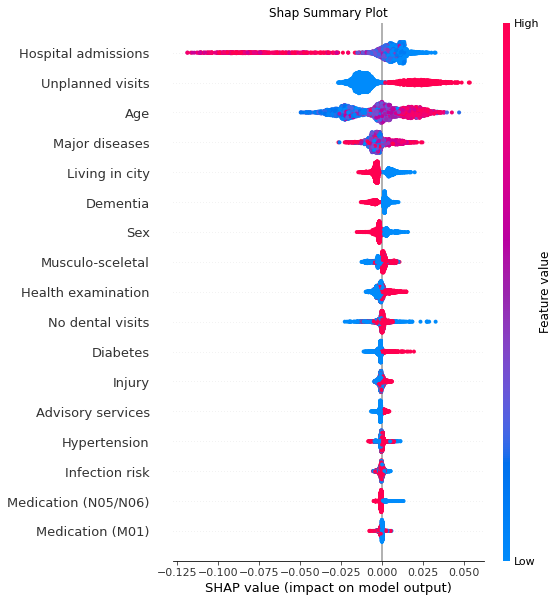  d: Mental health |
| 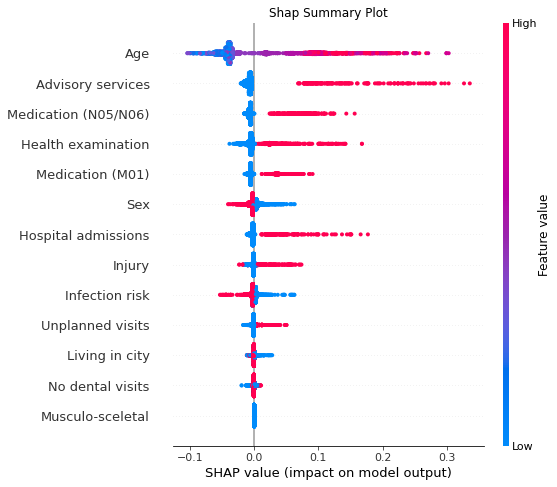  e: No major disease | 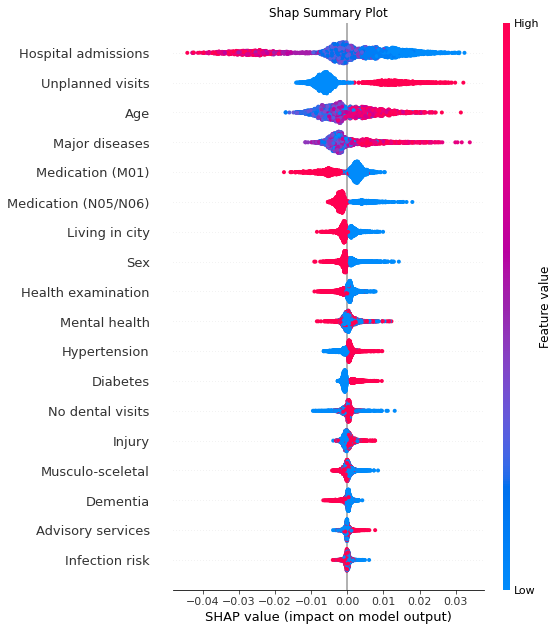  f: Elderly services customer |
| 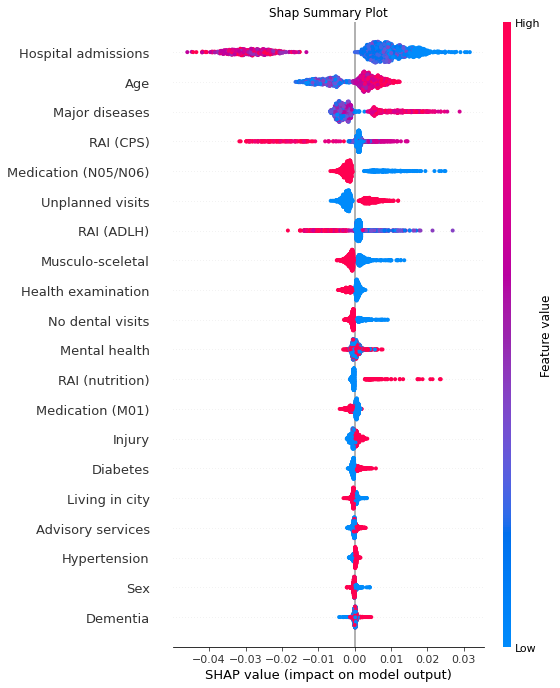  g: Home care customer |  |

Supplementary Table S4. XGBoost models hyperparameters

| **All participants** | **Dementia** |
| --- | --- |
| colsample_bytree: 0.8  gamma: 0  learning_rate: 0.01  max_depth: 6  scale_pos_weight: 3  subsample: 0.8 | colsample_bytree: 0.8  gamma: 1  learning_rate: 0.001  max_depth: 6  scale_pos_weight: 1  subsample: 1 |
| **Diabetes** | **Mental health or substance abuse** |
| colsample_bytree: 1  gamma: 1  learning_rate: 0.01  max_depth: 6  scale_pos_weight: 1  subsample: 0.8 | colsample_bytree: 1  gamma: 0  learning_rate: 0.001  max_depth: 6  scale_pos_weight: 3  subsample: 0.8 |
| **No major disease** | **Elderly services customer** |
| colsample_bytree: 0.8  gamma: 1  learning_rate: 0.01  max_depth: 6  scale_pos_weight: 1  subsample: 0.8 | colsample_bytree: 0.8  gamma: 1  learning_rate: 0.001  max_depth: 10  scale_pos_weight: 1  subsample: 0.8 |
| **Home care customer** |  |
| colsample_bytree: 0.8  gamma: 1  learning_rate: 0.001  max_depth: 6  scale_pos_weight: 1  subsample: 0.8 |  |
